# Non-Invasive Evaluation of Pulmonary Artery Stenosis in Congenital Heart Disease Patients Using Phase Contrast MRI

**DOI:** 10.1007/s13239-026-00843-z

**Published:** 2026-05-28

**Authors:** Rupak K. Banerjee, Gavin A. D’Souza, Shreyash M. Manegaonkar, Israel O. Ajiboye, Michael D. Taylor

**Affiliations:** 1 Department of Biomedical Engineering, University of Cincinnati, 593 Rhodes Hall, Cincinnati, OH 45221 USA; 2 Present Address: Department of Mechanical and Materials Engineering, University of Cincinnati, Cincinnati, OH USA; 3 The Heart Institute, Cincinnati Children’s Hospital Medical Center, Cincinnati, OH USA

**Keywords:** Pulmonary artery stenosis, Congenital heart disease, Functional diagnostic parameters, Phase contrast magnetic resonance imaging, *In vitro* experiments

## Abstract

**Background:**

Branch pulmonary artery (PA) stenosis is a significant congenital heart defect causing elevated pulmonary blood pressure, trans-stenotic pressure drop, and abnormal differential blood flow to the lungs. We propose combined pressure-flow diagnostic parameters, *pressure drop coefficient* (*CDP*) and *normalized energy loss* (*Ē*_*loss*_) for improved delineation of stenosis severity. In extension to our previous benchtop experimental study, we performed *in vitro* experiments using phase contrast magnetic resonance imaging (PC MRI) to evaluate the diagnostic parameters and stenosis severity non-invasively.

**Methods:**

Subject-specific branch PA test sections representing the main, left, and right PAs (MPA, LPA, and RPA) with a discrete LPA stenosis were manufactured from medical images using additive manufacturing. Three clinically-relevant stenosis severities, 70% area stenosis (AS), 80% AS, and 90% AS, were evaluated at 2 LPM cardiac output under pathophysiologic hemodynamics generated using a mock circulatory loop. 2D (all severities) and 3D (only 70% AS) PC MRI scans were performed to compute the velocity (or flow) and derived pressure drop across the PA test section. The CDP_LPA_ and ${\overline{E} }_{loss, LPA}$ were evaluated for the three stenosis severities using MRI measurements and compared against results from the benchtop study.

**Results:**

The CDP_LPA_ increased with an increase in LPA stenosis severity [70% AS: 11 (2D PC), 11.5 (3D PC); 80% AS: 34.6; 90% AS: 154.2]. These values resulted in an absolute difference of within 107 (2D PC) and 3 (3D PC) compared to the benchtop values. Interestingly, the CDP_LPA_ computed by all three methods (benchtop, 2D and 3D PC MRI) showed distinct and non-overlapping ranges for each stenosis severity [70% AS: 11.0–14.5; 80% AS: 34.6–60.7; and 90% AS: 154.2–261.6]. The ${\overline{E} }_{loss, LPA}$ (absolute) generally increased with an increase in LPA stenosis severity, except for 90% [70% AS: − 435 mJ per Q_LPA_ (2D PC), − 376 mJ per Q_LPA_ (3D PC); 80% AS: − 945 mJ per Q_LPA_; 90% AS: − 933 mJ per Q_LPA_ (Q_LPA_–LPA flow rate)].

**Conclusions:**

The CDP_LPA_ demonstrates an improved diagnostic capability over ${\overline{E} }_{loss, LPA}$ in accurately delineating unilateral PA stenosis severity. Further, PC MRI is a comprehensive and safe clinical modality for the non-invasive diagnosis of branch PA stenosis.

## Background

A stenosis in the branch pulmonary arteries (PA), clinically known as peripheral pulmonary stenosis, is a significant congenital heart defect having an estimated incidence of 836 per million live births [1–3]. The defect most often presents itself in conjunction with other congenital heart diseases (CHDs) such as tetralogy of Fallot (TOF) and truncus arteriosus; or genetic syndromes such as Williams, Alagille, or DiGeorge. It can also occur as a result of surgical procedures to correct CHDs [4–7]. Peripheral pulmonary stenosis may be present either as a discrete lesion or a diffuse narrowing in one (unilateral stenosis) or both (bilateral stenosis) branches of the pulmonary artery (PA) tree. Specifically, a residual unilateral PA stenosis, occurring after TOF repair, affects differential blood flow to the lungs resulting in impaired exercise capacity[8]. Additionally, it worsens pulmonary insufficiency and elevates proximal pressure causing RV overload [9, 10].

General guidelines for clinical diagnosis of PA stenosis include more than 20 mmHg pressure gradient across the stenosis, less than 35% differential flow to the affected lung, RV pressure load with two-third systemic pressure, and sometimes pulmonary valve regurgitation as an indirect marker [9]. Guided by the diagnosis, patients are treated with interventional therapy, including stenting or angioplasty to alleviate symptoms of the defect. Although these interventional procedures have shown to be effective in improving patient outcomes and quality of life, they are associated with procedural risks [4, 7, 11, 12]. It is important to diagnose stenosis severity accurately to minimize patient risk, and limit intervention to selected patients who require therapy. Also, reintervention is common in the clinical management of PA stenosis, which further warrants the need for accurate and continuous (longitudinal) monitoring of disease progression [11, 12].

### Comparison of Current Modalities with Proposed Indices for Assessing PA Stenosis

Clinical modalities including echocardiography, computed tomography (CT), cardiac catheterization, and cardiac magnetic resonance imaging (MRI) are standard test methods for the initial evaluation and follow-up of peripheral pulmonary stenosis [3, 13, 14].

*Echocardiography* is a primary non-invasive functional-anatomical modality, which is an advantage, for assessing PA stenosis but is fraught with limitations such as a) load dependent variations in flow impacting derived-pressure drop, b) inability to account for pressure recovery, c) measurement inaccuracies in branch pulmonary artery flow, and d) recirculating/vortical flow phenomenon. Furthermore, echocardiography is highly operator-dependent and can be inaccurate due to poor acoustic windows and angle-dependent Doppler measurements. Additionally, it lacks detailed anatomical resolution.

*CT Angiography* provides high-resolution, three-dimensional anatomical imaging of the pulmonary arteries, including distal branches. Although CT angiography is useful in detecting the presence of a stenosis [13], it lacks the ability to characterize the functional severity of the stenosis. Moreover, CT requires ionizing radiation and uses iodinated contrast, which poses allergy and risk to the kidney, particularly in pediatric populations. This causes it to be less suitable for repeated longitudinal (time) evaluation.

*Catheterization* is currently the primary standard-of-care clinical modality providing direct measurement of pressure drop. It enables clinicians to distinguish physiologically significant lesions from insignificant ones and supports interventional decision-making. However, catheterization is associated with procedural risk to pediatric patients due to it being invasive modality[15, 16]. Catheterization, often requires sedation in pediatric patients, is technically challenging in small or tortuous vessels. Additionally, catheterization for functional measurement does not provide anatomical detail and must be interpreted alongside imaging studies.

*Magnetic Resonance Imaging (MRI)* is a non-invasive, radiation-free tool for longitudinal assessment of PA stenosis in pediatric patients. MRI provides higher contrast of soft-tissues, enabling detailed anatomical visualization of the PA and surrounding structures. Advanced techniques such as phase-contrast MRI (PC-MRI) and 3D MRI for different time-points of a cardiac cycle allows quantification of important hemodynamic parameters. In other words, MRI can provide both functional and anatomical assessment sequentially in one sitting. However, MRI scan times are longer than echocardiography or CT. It often requires sedation in children or uncooperative patients, and its availability is limited due to cost concerns.

Conventional 2D PC MRI, which provides time-resolved through-plane (one-directional) velocity (and flow) data, has been used as a clinical standard of care to assess flow across obstructive lesions [8, 17]. Further, the feasibility of obtaining non-invasive pressure drop across a coronary stenosis using 2D PC MRI has been assessed in a pre-clinical and clinical setting [18]. Alternatively, 3D PC MRI offers the ability to measure and visualize the temporal evolution of complex flow field in a 3D space [19]. Our group has previously tested the feasibility of using 3D PC MRI to evaluate the non-invasive pressure field (and pressure drop) across a PA stenosis using retrospective clinical data [20]. Despite its theoretical advantages over 2D PC MRI, 3D PC MRI is still used occasionally in routine clinical practice.

MRI and echocardiography are used to assess the pathophysiology of pulmonary blood flow to the lungs in the presence of a stenosis[8], while providing anatomical details of the vessel [21, 22]. The utility of these two non-invasive modalities has been extended to evaluate derived pressure drop using simplified Bernoulli’s equation from velocity data. To the author’s knowledge, assessment of these modalities in presence of concomitant branch and stenotic geometries using either more accurate modified Bernoulli’s equation with viscous loss or Navier-Stokes equation has not been reported.

### Proposed Diagnostic Index

Current diagnostic endpoints of PA stenosis are discrete -- based on either flow or pressure drop. They do not capture the complexity of branched flow from the main pulmonary artery (MPA) with asymmetric pressure drops and flow distributions. We evaluated the proposed diagnostic parameters, *pressure drop coefficient* (*CDP: ratio of trans-stenotic pressure drop to the proximal dynamic pressure* [23] *- a function of square of velocity-*and *normalized energy loss (Ē*_*loss*_*: ratio of the total energy loss due to a stenosis to the flow across the stenosis).* These two indices incorporates both pressure drop and flow in their formulation. Using a benchtop experiments and patient-specific vascular phantoms [24], we tested these two indices in a unilateral left PA (LPA) stenosis having three anatomical severities – 70% area stenosis (AS), 80% AS, and 90% AS. CDP showed a wider range [24] to delineate the severity of PA stenosis, because of square of velocity in its denominator. Therefore, CDP has the potential to aid in improved assessment of the PA stenosis.

2D and 3D PC MRI were used in the current study to compute the non-invasive pressure drop across the unilateral LPA stenosis from velocity data using analytical approach. Subsequently, we evaluated CDP and Ē_loss_ to delineate the functional severity of the 70%, 80%, and 90% AS. Subject-specific vascular phantoms were used to mimic the different severities of LPA stenosis within a mock circulatory loop (MCL) that generated pathophysiologic PA hemodynamics. The utility of the MRI-based methodology for diagnosing a unilateral LPA stenosis has been assessed by comparing the results from this study with sensor-based direct pressure-flow measurements reported in our previous benchtop experimental study [24].

The present study is *novel* because it uses a non-invasive modality (MRI) to evaluate combined pressure-flow based CDP and Ē_loss_ within a unilateral LPA stenosis of varying severities. The *significance* of this pre-clinical study is that it will lead to future clinical trials for the evaluation of CDP and Ē_loss_.

## Methods

**Fig. 1:**
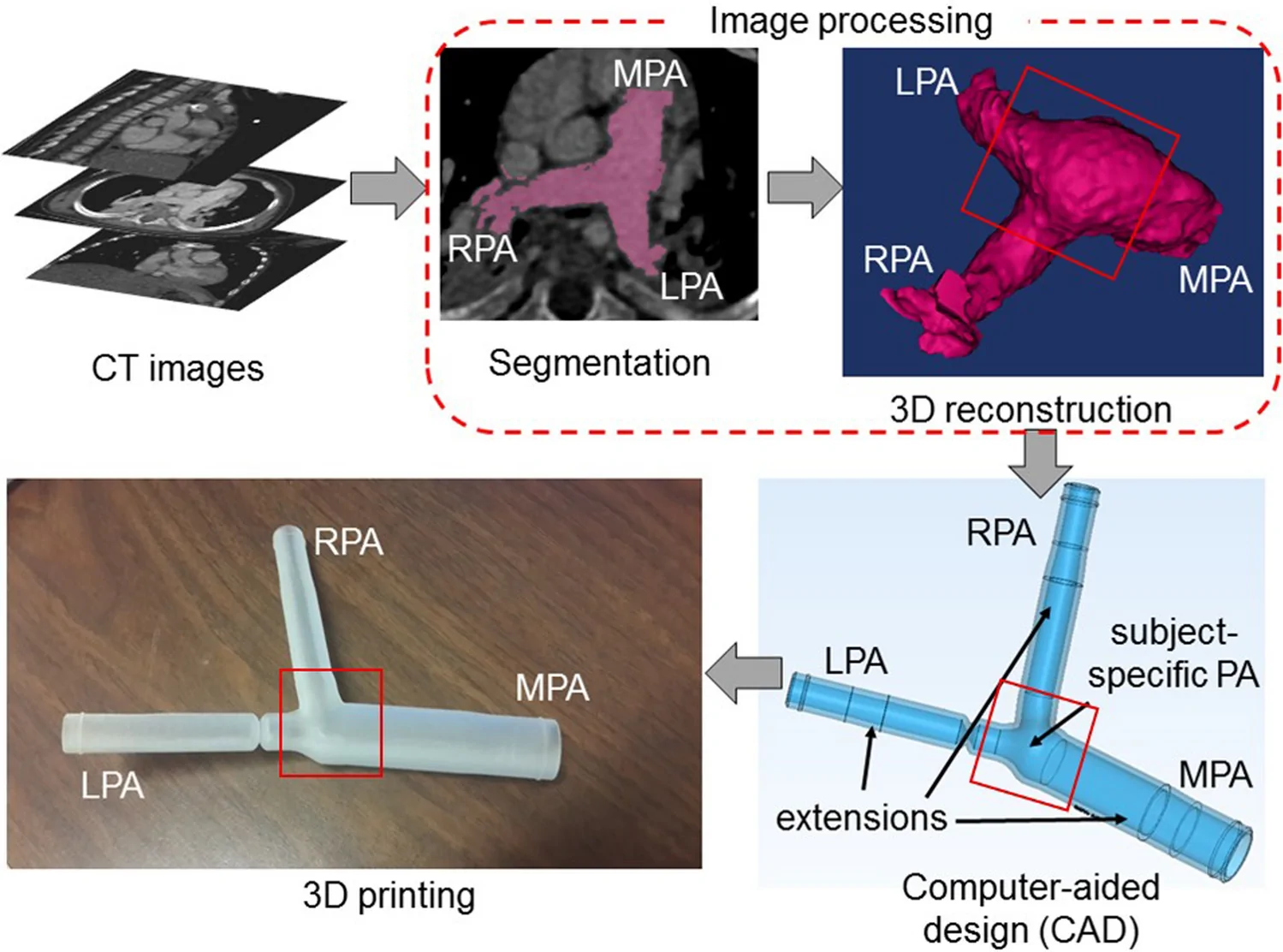
Diagrammatic representation of the manufacturing process for a 3D subject-specific pulmonary artery test section (*MPA* main pulmonary artery, *LPA* left pulmonary artery, *RPA* right pulmonary artery)

The *in vitro* setup used for conducting PC MRI experiments is similar to that used in our benchtop experimental study [24]. A detailed description of the manufacturing process used for developing the subject-specific vascular phantom (Fig. 1) has been previously reported [25]. Thus, only a brief description of the experimental setup with key details is provided below.

### Experimental Setup

The pulmonary vascular anatomy of an 8-month old female subject, including the native main, left, and right PAs (MPA, LPA, and RPA), was reconstructed from a computed tomography (CT) angiogram using standard image segmentation and 3D reconstruction. To mimic a unilateral PA stenosis, a central discrete narrowing [5] was incorporated in the LPA using computer-aided design (CAD). Subsequently, additive manufacturing (3D printing) was used to obtain a physical representation of the subject-specific PA test section from the CAD model.

**Fig. 2:**
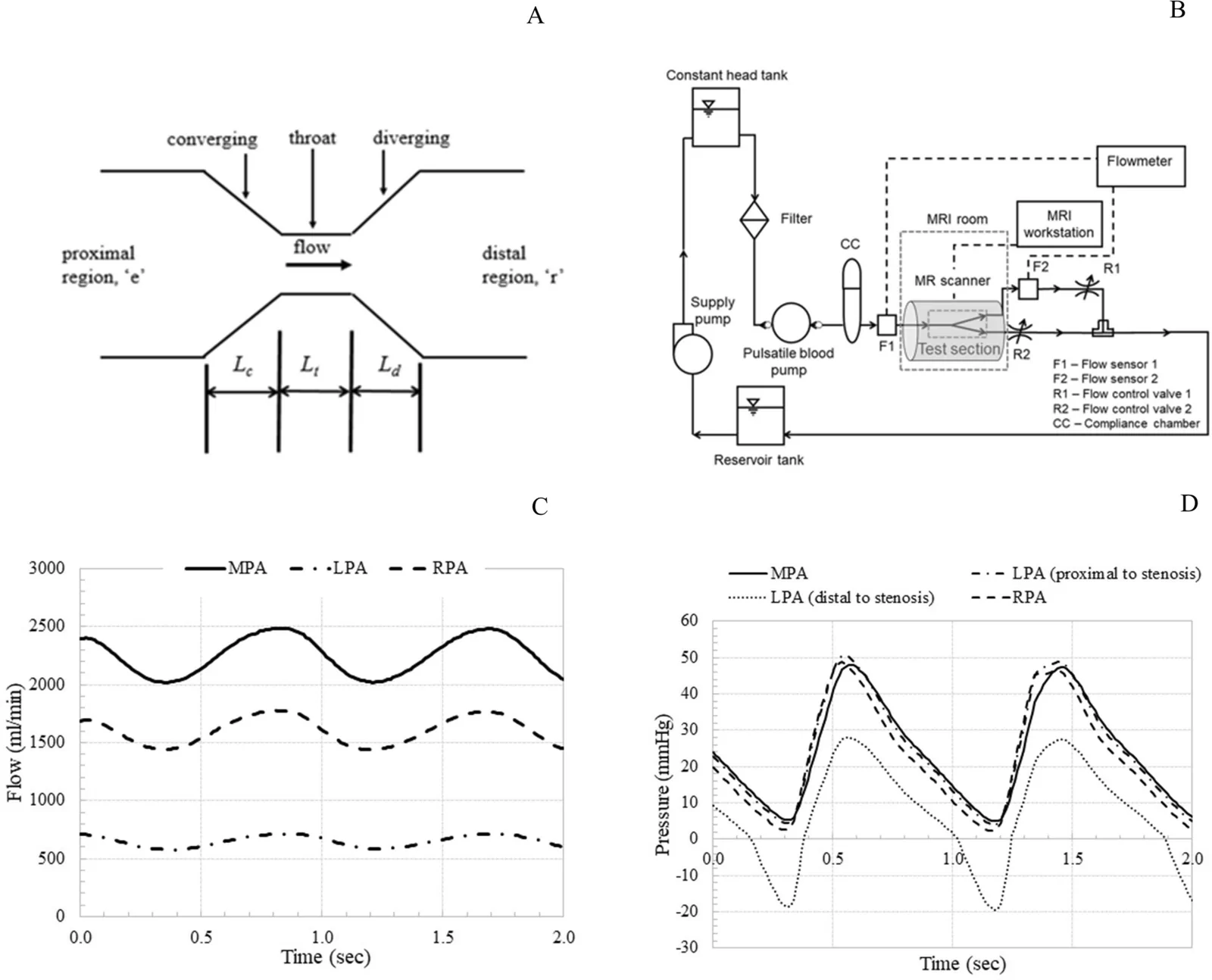
**A** A schematic of the stenosis (*L*_*c*_ converging length, *L*_*t*_ throat length, *L*_*d*_ diverging length), **B** A schematic of the MRI experimental mock circulatory flow loop (MCL), Representative experimental pulmonary artery **C**) flow, and **D**) pressure pulses for an average of 2 LPM flow rate

*Test section*. A typical geometry of the LPA stenosis, comprising of the converging, throat, and diverging regions, is shown in Fig. 2A. The regions proximal and distal to the stenosis are denoted by ‘e’ and ‘r,’ respectively. Based on clinical evidence [26], three anatomical severities of the LPA stenosis were evaluated: a) mild (~ 70% area stenosis (AS)), b) moderate (~ 80% AS), and c) severe (~ 90% AS). The geometric dimensions corresponding to the different stenosis severities are provided in Table 1. While only a single representative test section is shown in Fig. 1, three test sections representing the different stenosis severities were manufactured and used for the experiments. The section shown within the red box that includes the MPA, LPA, and RPA is used to develop subject-specific 3D vascular phantom based on the segmentation of CT images. The final 3D-printed model includes modifications, such as inlet/outlet extensions and interface connectors, to facilitate integration with the MCL.

**Table 1:** Geometric dimensions of the three different stenoses

Stenosis severity	*L*_*c*_*, L*_*t*_*, L*_*d*_ (mm)	*d*_*e*_ = *d*_*r*_ (mm)	*d*_*t*_ (mm)
Mild − 70% AS	2	8.8	4.78
Moderate − 80% AS	2	8.8	3.74
Severe − 90% AS	2	8.8	2.96
Area of native vessels: A_MPA_ = 220 mm^2^; A_LPA_ = 61 mm^2^; A_RPA_ = 70 mm^2^			

*L*_*c*_ converging length, *L*_*t*_ throat length, *L*_*d*_ diverging length, *d*_*e*_ proximal LPA diameter*, d*_*t*_ stenosis throat diameter*, d*_*r*_ distal LPA diameter, *x* axial direction, *r* radial direction

*Mock Circulatory Loop (MCL)*. Pathophysiologic hemodynamics within the stenotic PA test section were simulated using an MCL [24, 27–29], shown in Fig. 2B. Except for the PA test section, all components of the MCL were placed outside the MR scanner room (Fig. 2B). The pump, valves and fittings, instrumentation and data acquisition systems were not MR compatible and had to be placed outside the MR scanner room. This was necessary to avoid interference of magnetic field of the MR scanner with the metallic components of the pump, valves and fittings, instrumentation and data acquisition systems. Therefore, the blood analog fluid (BAF) had to travel through an extended tubing of additional ~ 25 ft due to MR scanner compatibility constraints, resulting in pressure and flow damping. The PA test section was secured to the MRI scanner bed and surrounded by saline bags. The saline bags were used to reduce artifacts due to the volume of air surrounding the test section. After securing the test section on the scanner bed, the bed was moved such that the test section was centrally located within the magnet bore. The 2D and 3D PC MRI scans were performed on the stenotic PA test section using a 1.5 T MRI scanner (*Ingenia*, Philips) and an 8-channel radiofrequency (RF) coil receiver.

Non-Newtonian BAF [24, 27–29] with dynamic viscosity, μ = 3.46 cP (at infinite shear rate), and density, ρ = 1057 kg/m^3^, was circulated through the test section using a pulsatile blood pump. Vascular compliance was simulated using air-filled compliance chambers, while the flow through each branch was controlled independently using flow control valves. An ultrasound Doppler in-line flow sensor F1 (ME−13PXN, Transonic Systems, Inc.) was placed distal to the compliance chamber (Fig. 2B) and recorded the MPA flow rate (or CO). A second ultrasound Doppler clamp-on flow sensor F2 (ME−9PXL, Transonic Systems, Inc.) was placed at the exit of the test section in the LPA branch (Fig. 2B) and recorded the LPA flow rate. F1 and F2 were calibrated up to an accuracy of ± 4% and ± 10%, respectively. The RPA flow rate was computed indirectly based on flow balance within the PA test section. Both flow sensors were coupled to a digital flowmeter console (TS410, Transonic Systems, Inc.) to acquire and display flow rates.

*Pressure-Flow input*. Representative experimental pulmonary artery flow and pressure pulses are reported in Figures 2C and 2D for an average of 2 LPM flow rate. The cardiac cycle duration (T_cycle_) was ~ 0.9 s (T_systole_ ~ 0.4 s, T_diastole_ ~ 0.5 s). This benchtop experiment was conducted without the extended ~ 25 ft tubing. Similar to the benchtop experiments [24], the p_MPA_ was maintained between 17 mmHg and 26 mmHg. To achieve this desired pressure at the inlet of the test section within the MR scanner, a higher pressure was required at the outlet of the pulsatile blood pump, located outside of the MR scanner room. This was necessary to overcome the pressure loss due to viscous damping and added compliance along the length (~25 ft.) of the tubing transporting BAF from the pump to the test section. Since the pressure within the test section could not be monitored simultaneously during the MR experiments, a pressure calibration test was conducted using the modified benchtop system having additional ~ 25 ft of tubing to pre-determine the required realistic pump outlet pressure that is applicable for MR experiments. The p_MPA_ was varied between 18 and 25 mmHg at a fixed CO of 2 LPM and a fixed (arbitrary) ${Q}_{LPA}/{Q}_{MPA}$ of 28%, while the corresponding pump outlet pressure was recorded for the modified benchtop experiments. A constant pressure difference (loss) of 9 mmHg was observed for all the p_MPA_ values between the benchtop [24] and the modified benchtop experiment having additional ~ 25 ft tubing. In other words, during MR experiments the pump was operated 9 mmHg higher than the operating pressure used in the benchtop experiment, as also previously reported [24].

The flow (Fig. 2C) and pressure (Fig. 2D) values were first averaged over a cardiac cycle to obtain mean values, respectively. Subsequently, a statistical average of such mean values of the three experimental dataset was computed. The averaged pressure-flow data was used to obtain pressure drop and flow rates (Q).

A cardiac output (CO) of 2 L/min was selected. Clinically, 2 L/min CO is near the lower end of the normal range for pediatric patients, as reported in population studies [30]. This level of CO is also commonly observed in congenital heart disease patients with low-output states, causing it to be relevant and representative choice for our study. Furthermore, it also matched the conditions for which comprehensive benchtop data were available, allowing direct comparison with MRI results. The study was also limited by the specification and sizing of the currently available Harvard apparatus pump (Model 1423). It may be noted that a higher flowrate of 5 L/min Harvard apparatus pump is designed for systemic circulation of left heart in adults, whereas a lower flowrate of 2 L/min Harvard apparatus pump (Model 1423) is suited for reduced CO of right heart as in pediatric patients or for coronary circulation in adults.

### 2D PC MRI

**Fig. 3:**
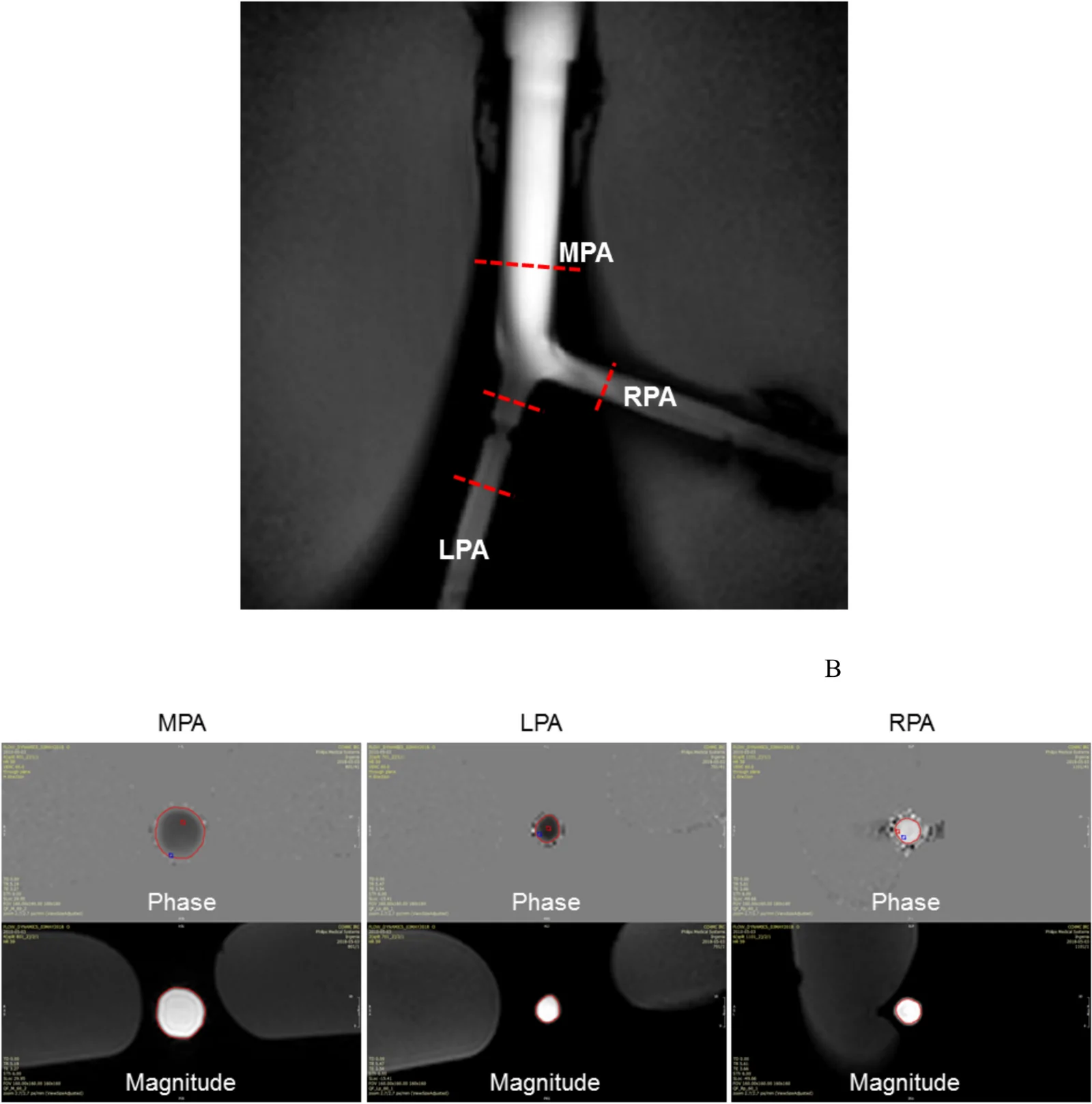
**A** A representative 2D CMR image of the PAs. Red dashed lines indicate the location of the planes at which 2D PC MR images were acquired, **B** Representative 2D PC MR images (magnitude and phase) showing a cross-sectional view of the PAs. Contours marked in red represent the area of the PA through which flow is observed

2D PC MRI was performed at cross-sectional planes placed perpendicular to the MPA, LPA, and RPA. A representative cardiac MR (CMR) image of the longitudinal view of the MPA, LPA, and RPA, obtained in the long-axis plane of the PA test section, is shown in Fig. 3A. A total of four cross-sectional planes (red dashed lines in Fig. 3A) were used – one each at the MPA and RPA, and one each in the proximal and distal regions of the LPA stenosis. At each plane, cine PC MR images were acquired at 20 time-points over the simulated cardiac pulse. Details of the 2D PC MR imaging sequence are provided in Table 2. The aliasing velocity (VENC), echo time (TE), and repetition time (TR) were selected to obtain optimal image resolution.

**Table 2:** Details of the 2D PC MR imaging sequence

Parameter	Value
Number of phases	20
Slice thickness [mm]	6
Field of view [mm^2^]	160 × 160
Spatial resolution [mm]	1 × 1 mm^2^
Temporal resolution [ms]	51
Flip angle [degrees]	12
Aliasing velocity, VENC [cm/s]	60–150
Echo time, TE [ms]	~ 3.27
Repetition time, TR [ms]	~ 5.19
Number of excitations	4

Representative phase and magnitude images for the MPA, LPA, and RPA at a specific time point are shown in Fig. 3B. Time-resolved flow rate and velocity data of the PAs were obtained from the 2D PC MR images using a medical image-processing software (*cvi42*, Circle Cardiovascular Imaging). The white pixels in the magnitude images (Fig. 3B) represent the PA flow domain. Using semi-automated thresholding, contours (marked in red in Fig. 3B) were drawn around the periphery of the PA flow domain on all the image slices. Flow rate and velocity data over the cardiac cycle were computed from the intensity values of the pixels enclosed by the contours.

### 3D PC MRI

**Fig. 4:**
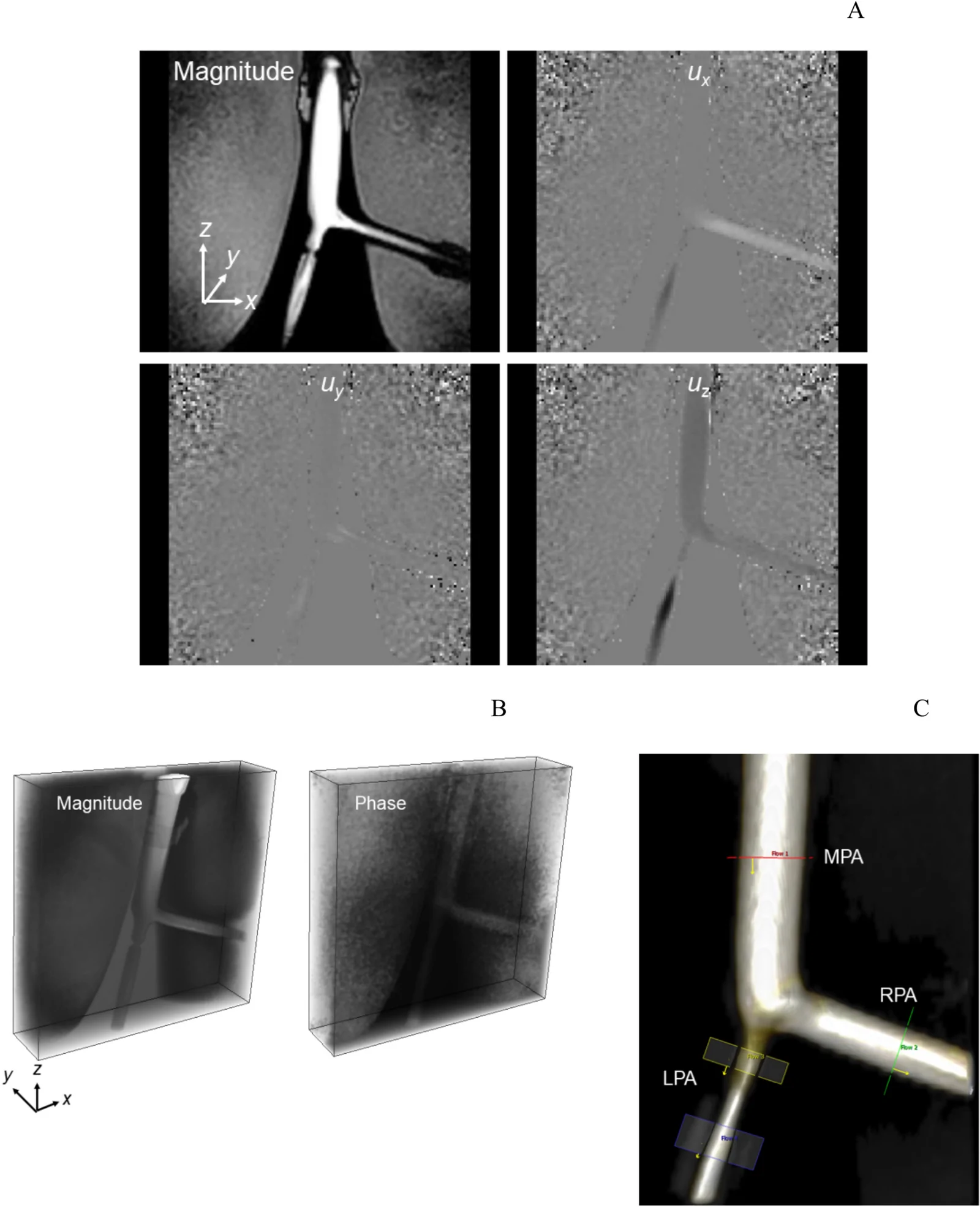
**A** A representative 3D PC MRI dataset comprising of the magnitude image and three-directional phase images containing velocity information (*u*_*x*_, *u*_*y*_, *u*_*z*_), **B** 3D volume rendered blocks showing the magnitude and phase data of the PA fluid domain obtained from 3D PC MRI at a specific time point, and **C** A representative 3D rendered volume of the PA flow domain acquired using 3D PC MRI along with cross-sectional cut planes for flow and velocity measurements

3D PC MRI data was acquired for 20 cardiac phases yielding a 4D PC dataset. Details of the 3D PC MR imaging sequence are provided in Table 3. The PC images were acquired in the anterior to posterior direction (i.e. coronal or y plane; shown in Fig. 4A: *Magnitude* image), and each image was subsequently reconstructed (using isotropy) in three orthogonal planes (coronal or y, sagittal or x, and axial or z; shown in Fig. 4A: *Magnitude* image) to obtain three-directional phase images containing velocity data.

**Table 3:** Details of the 3D PC MR imaging sequence

Parameter	Value
Number of slices	40
Number of phases	20
Voxel dimension [mm^3^]	1.5 × 1.5 × 1.5
Temporal resolution [ms]	51
Flip angle [degrees]	8
Aliasing velocity, VENC [cm/s]	130–170
Echo time, TE [ms]	2.27
Repetition time, TR [ms]	4.22
Number of excitations	3

Figure 4A shows a representative 3D PC MRI dataset consisting of the magnitude and phase images containing three-directional velocity data, i.e., *u*_*x*_, *u*_*y*_, and *u*_*z*_*,* at a specific time point of the cardiac cycle. Each dataset was subsequently rendered into a 3D volume (*cvi42*, Circle Cardiovascular Imaging) representing the PA flow domain containing magnitude and phase information as shown in Fig. 4B. After segmenting the fluid volume within the PA test section, cross-sectional cut planes were placed perpendicular to the MPA, LPA, and RPA, similar to the planes in the 2D PC MRI scan. Fig. 4C shows a representative 3D rendered fluid volume of the PAs along with cut planes in the MPA, RPA, and LPA. Time-resolved flow rate and velocity were obtained at the cross-sectional planes using the same threshold-based image processing technique described for the 2D PC MRI scan.

### Experimental Procedure

**Fig. 5:**
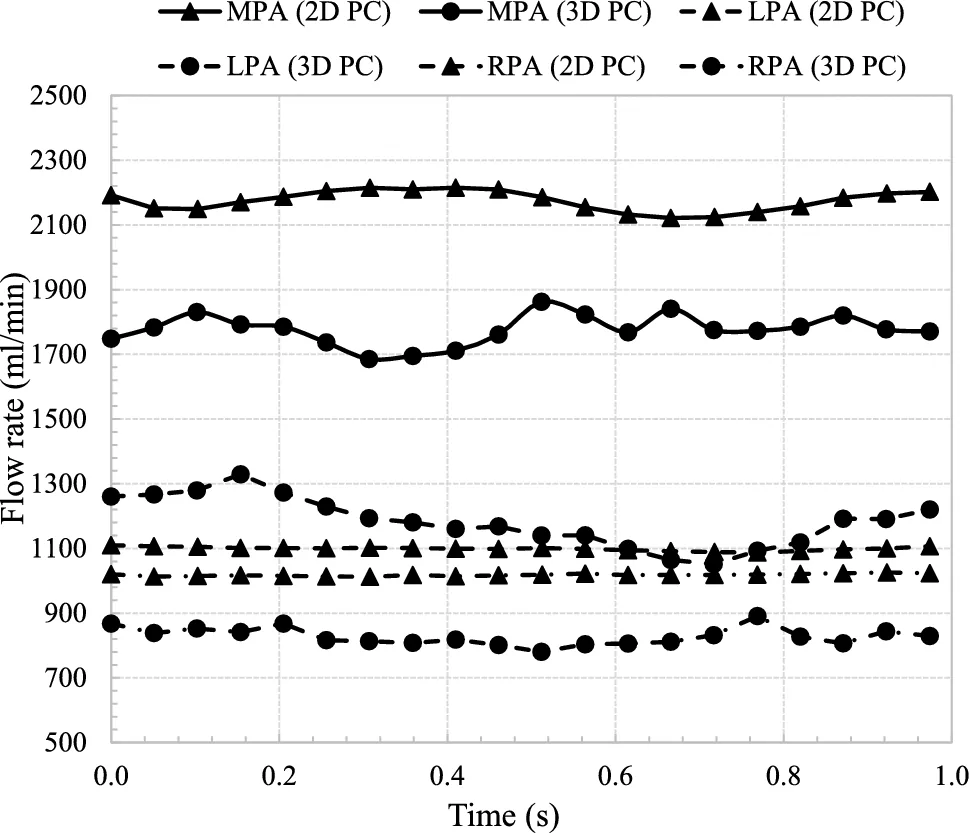
Representative flow rate pulses representing pathophysiologic conditions of the pulmonary vasculature acquired using PC MRI

The pathophysiologic LPA flow split, ${Q}_{LPA}/{Q}_{MPA}$, corresponding to the 70%, 80%, and 90% AS LPAs at specific cardiac outputs (COs) was evaluated in our benchtop experimental study using pressure drop-flow rate (Δp-Q) characteristics derived from clinical data [24]. The PC MRI experiments were designed based on our prior understanding of the unilateral LPA stenosis pathophysiology. The target pathophysiologic ${Q}_{LPA}/{Q}_{MPA}$ values set for the 70%, 80%, and 90% AS cases were 47%, 36%, and 21%, respectively. Further, the mean (time-averaged) MPA pressure, ${p}_{MPA}$, was maintained between 17 mmHg and 26 mmHg for the different stenosis severities [24]. 2D PC MRI data was acquired for all the three LPA stenosis severities, i.e., 70%, 80%, and 90% AS, while 3D PC MRI was acquired for the 70% AS only as a means of an initial evaluation of the novel technology. Representative pulmonary flow pulses, recorded using the MRI scanner, are shown in Fig. 5.

### Non-invasive Pressure Drop

Using the flow rate, Q, and velocity, u, obtained at the cross-sectional planes, the mean pressure drop from the MPA to the LPA, distal to the stenosis, ${\Delta p}_{MPA-LPA}$, was computed using the mass, momentum, and energy conservation equations.

#### Pressure Drop Across the Converging Region

Applying mass and momentum conservation to the converging region of the stenosis (Fig. 2A), the mean pressure drop (in Pa) across the converging region [31, 32] was computed as:

1a $${\Delta p}_{c}=\frac{128\mu {L}_{c}{Q}_{LPA}{I}_{s}}{\pi {d}_{e,LPA}^{4}}+\beta \frac{1}{2}\rho {u}_{e,LPA}^{2}\left[{\left(\frac{{A}_{e,LPA}}{{A}_{t,LPA}}\right)}^{2}-1\right]$$

where $${I}_{s}={\int}_{0}^{1}\frac{{d}_{e,LPA}^{4}}{{\left[{d}_{e,LPA}-\frac{x}{{L}_{c}}\left({d}_{e,LPA}-{d}_{t,LPA}\right)\right]}^{4}}d\left(\frac{x}{{L}_{c}}\right)$$

and $$\beta =\frac{1}{{u}_{e,LPA}^{2}{A}_{e,LPA}}\int {u(r)}^{2}dA$$

μ (Pa.s) is the dynamic viscosity, u (m/s) is the mean velocity, ρ (kg/m^3^) is the fluid density,* L* (m) is the length, *d* (m) is the diameter, *A* (m^2^) is the area, and *x* (m) is the axial distance from the start of the converging region (subscripts ‘*e, LPA*,’ ‘*t, LPA*,’ and ‘*r, LPA*’ represent the proximal, throat, and distal regions of the LPA stenosis, respectively). Considering a lower Reynolds number (Re; peak Re ~ 1400) and therefore, assuming a parabolic velocity profile at any instant in time in the proximal region of the stenosis, the momentum flux correction factor, $\beta =\frac{4}{3}$ [31]. The trapezoidal integration method was used to compute ${I}_{s}$. A detailed derivation of Equation 1a using the mass and momentum conservation equations is provided in Appendix 1.

#### Pressure Drop Across the Throat Region

Applying momentum conservation to the throat region of the stenosis, the mean pressure drop (in Pa) across the throat region [31, 32] was computed as:

1b $${\Delta p}_{t}=\frac{128\mu {L}_{t}{Q}_{LPA}}{\pi {d}_{t,LPA}^{4}}$$

#### Pressure Recovery Across the Diverging Region

Distal to the stenosis, there is pressure recovery (i.e. increase in pressure) due to flow reattachment. Applying momentum conservation between the stenosis throat region and the distal region of the stenosis, the mean pressure recovery (in Pa) distal to the stenosis [31, 32] was computed as:

1c $${\Delta p}_{r}=k\rho {u}_{t,LPA}^{2}\frac{{A}_{t,LPA}}{{A}_{r,LPA}}\left(1-\frac{{A}_{t,LPA}}{{A}_{r,LPA}}\right)$$

where ${A}_{r,LPA}={A}_{e,LPA}$ and factor *k* = 0.833 for physiological flows[31].

#### Pressure Drop Due to Branching

The mean pressure drop (in Pa) due to PA branching [33, 34] was computed as:

1d $${\Delta p}_{br}={K}_{br}\frac{1}{2}\rho {u}_{MPA}^{2}$$

where the branch loss coefficient, ${K}_{br}$, corresponding to the 70%, 80%, and 90% AS was 2.4, 1.35, and 0.09, respectively[33, 34].

#### Overall Pressure Drop

Therefore, the resultant mean pressure drop (in Pa) from the MPA to the LPA, distal to the stenosis was computed from the 2D and 3D PC MRI scans using Eqns. 1a – 1d as:

2 $${\Delta p}_{MPA-LPA}={\Delta p}_{c}+{\Delta p}_{t}+{\Delta p}_{br}-{\Delta p}_{r}$$

The ${\Delta p}_{MPA-LPA}$ and the corresponding pathophysiologic ${Q}_{LPA}$ (or ${u}_{LPA}$) values obtained using the MRI experiments were subsequently used to compute the functional diagnostic parameters defined below.

### Diagnostic Parameters

The proposed diagnostic parameters, *pressure drop coefficient* (CDP_LPA_) and *normalized energy loss* (${\overline{E} }_{loss, LPA}$) for evaluating the (functional) severity of LPA stenoses are defined below.

#### Pressure Drop Coefficient

CDP_LPA_ is defined as the ratio of the pressure drop across the LPA stenosis to the dynamic pressure proximal to the stenosis [23].

3 $${CDP}_{LPA}=\frac{{\Delta p}_{MPA-LPA}}{0.5\times \rho \times {{u}_{e,LPA}}^{2}}$$

#### Normalized Energy Loss

The ${\overline{E} }_{loss, LPA}$ (J per ${Q}_{LPA}$) is defined as the ratio of energy loss in the LPA (J) to the LPA flow rate (m^3^/s).

4 $${\overline{E} }_{loss, LPA}=\frac{{E}_{loss, LPA}}{{Q}_{LPA}}$$

where ${E}_{loss,LPA}$ (J) can be defined as the sum of the difference in pressure-flow and kinetic energies between the LPA and MPA (Lee et al, 2013), i.e., ${E}_{loss, LPA}=\left({p}_{r,LPA}-{p}_{MPA}\right){Q}_{LPA}+\frac{1}{2}\rho \left({{u}_{e,LPA}}^{2}-{{u}_{MPA}}^{2}\right){Q}_{LPA}$.

## Results

The hemodynamics of stenotic PAs, evaluated non-invasively using MRI, are described in this section. Specifically, the flow rates and proposed diagnostic parameters, CDP_LPA_ and ${\overline{E} }_{loss, LPA}$, evaluated using MRI are compared with data from our previous experimental study involving a benchtop test system [24]. Data referenced from this previous study has been labeled and referred to as ‘benchtop’ results hereafter.

### Flow Rate Comparison

The $${Q}_{MPA}$$, $${Q}_{LPA}$$, and $${Q}_{RPA}$$, evaluated using PC MRI were compared with those recorded directly by the flowmeter during the *in vitro* experiments to ensure correct implementation of the PC MRI technique. Tables 4 and 5 show the comparison of the flowmeter recordings with the flow rates evaluated using 2D and 3D PC MRI, respectively. The flow rates recorded using the flowmeter (approximately) represent the desired values that were set based on the pathophysiologic LPA flow splits ($${Q}_{LPA}/{Q}_{MPA})$$ corresponding to a specific stenosis severity [24]. The relative error (= $$\left[\frac{\left|{Q}^{flowmeter}-{Q}^{MRI}\right|}{{Q}^{flowmeter}}\right]\times 100$$) in the PC MRI measurements with respect to the flowmeter recordings are specified in parenthesis alongside the PC MRI flow rate values (Tables 4 and 5). The relative error in 2D PC MRI flow rates was within 6%, 19%, and 16% for the 70%, 80%, and 90% AS, respectively (Table 4). Therefore, the 70% AS produced the least error in flow rate compared to the 80% and 90% AS. The relative error in 3D PC MRI flow rates was within 19% for the 70% AS. Overall, the 3D PC MRI measurements showed a relatively higher error in flow rate values compared to 2D PC MRI measurements for the 70% AS. A 20% relative error for all the stenosis severities was considered reasonable for the current study [35].

**Table 4:** Mean PA flow rate obtained using the MCL flowmeter and PC MRI measurements

LPA stenosis severity	Flowmeter			2D PC MRI			
	${Q}_{MPA}$ (ml/min)	${Q}_{LPA}$ (ml/min)	${Q}_{RPA}$ (ml/min)	${Q}_{MPA}$ (ml/min)	${Q}_{LPA}$ (ml/min)	${Q}_{RPA}$ (ml/min)	% error in continuity
70% AS	2200	1037	1163	2174 (*1%*)	1018 (*2%*)	1099 (*6%*)	2.6
80% AS	2180	813	1367	2053 (*6%*)	826 (*2%*)	1109 (*19%*)	5.8
90% AS	2150	454	1696	2090 (*3%*)	384 (*16%*)	1789 (*6%*)	3.9

Italicized numbers in the brackets represent the % difference between the PC MRI and flowmeter values

**Table 5:** Flow rates obtained using 3D PC MRI and the MCL flowmeter

LPA stenosis severity	Modality	${Q}_{MPA}$ (ml/min)	${Q}_{LPA}$ (ml/min)	${Q}_{RPA}$ (ml/min)	% error in continuity
70% AS	Flowmeter	2200	1037	1163	–
	3D PC MRI	1771 (*19%*)	927 (*11%*)	1207 (*4%*)	20

Italicized numbers in the brackets represent the % difference between the PC MRI and flowmeter values.

Further, a check for conservation of mass (i.e., continuity) between the inflow (MPA) and outflows (LPA and RPA) was established using the PC MRI flow rates. The error in continuity (= $\left[\frac{\left|{Q}_{MPA}-\left({Q}_{LPA}+{Q}_{RPA}\right)\right|}{{Q}_{MPA}}\right]\times 100$) was within 5.8% for the 2D PC MRI data (Table 4) and 20% for the 3D PC MRI data (Table 5). After assessing the flow balance in the PC MRI data, the pathophysiologic ${Q}_{LPA}/{Q}_{MPA}$ and diagnostic parameters were evaluated.

### Pathophysiologic LPA Flow Split

The pathophysiologic ${Q}_{LPA}/{Q}_{MPA}$, computed using 2D PC MRI, is reported in Table 6. An increase in LPA stenosis severity lead to a decrease in the pathophysiologic ${Q}_{LPA}/{Q}_{MPA}$. At a CO of 2 LPM, the pathophysiologic ${Q}_{LPA}/{Q}_{MPA}$ was 47% for the 70% AS. This value is in accordance with the benchtop value (Table 6). In contrast, 3D PC MRI produced a 11% ($=\left[\frac{\left|{{[Q}_{LPA}/{Q}_{MPA}]}_{benchtop}-{Q}_{LPA}/{Q}_{MPA}\right|}{{{[Q}_{LPA}/{Q}_{MPA}]}_{benchtop}}\right]\times 100;$ here ${Q}_{LPA}/{Q}_{MPA}$ without the subscript is computed from either 2D or 3D PC MRI data) higher pathophysiologic ${Q}_{LPA}/{Q}_{MPA}$ (= 52%) for the 70% AS. When the stenosis severity increased to 80% AS, the MRI-based pathophysiologic ${Q}_{LPA}/{Q}_{MPA}$ was 40%, and was 12% higher than the benchtop value (Table 6). The MRI-based pathophysiologic ${Q}_{LPA}/{Q}_{MPA}$ for the 90% AS was 18%, resulting in a 13% lower value than the benchtop value (Table 6). This difference in MRI-based pathophysiologic ${Q}_{LPA}/{Q}_{MPA}$ with benchtop data is expected to influence the diagnostic parameters discussed below.

**Table 6:** Pathophysiologic LPA flow split (${Q}_{LPA}/{Q}_{MPA}$) and diagnostic parameters obtained using flowmeter and PC MRI

LPA stenosis severity	${Q}_{LPA}/{Q}_{MPA}$(%)		CDP_LPA_		${\overline{E} }_{loss, LPA}$ (mJ per ${Q}_{LPA}$)	
	Benchtop	2D PC MRI	Benchtop	2D PC MRI	Benchtop	2D PC MRI
70% AS	47	47	14.5	11.0	− 501.9	− 434.6
80%AS	36	40	60.7	34.6	− 1247.6	− 945.0
90%AS	21	18	261.6	154.2	− 1934.5	− 932.5

### *Pressure Drop Coefficient (CDP*_*LPA*_*)*

**Fig. 6:**
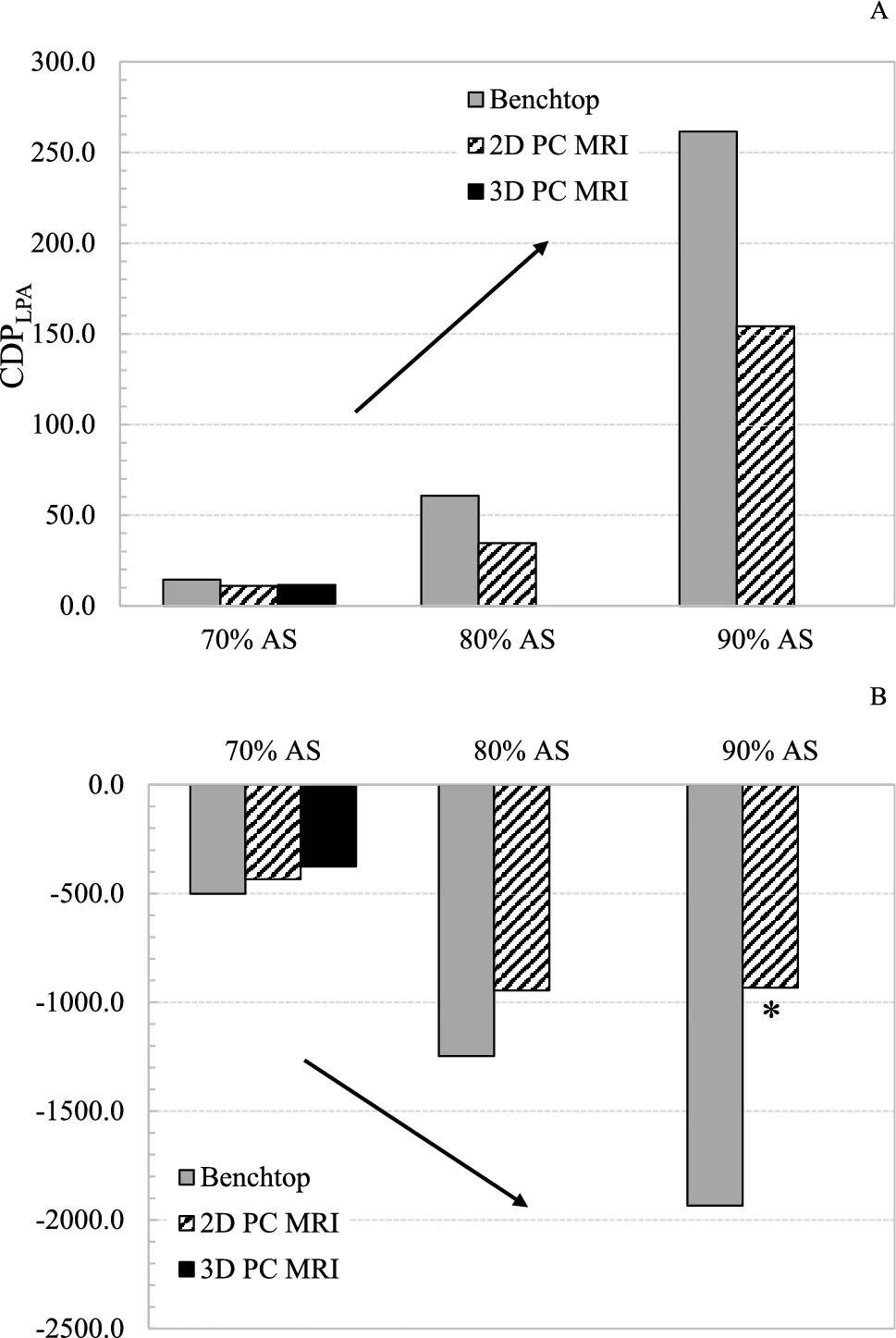
Bar graphs showing the comparison of the functional diagnostic parameters obtained using PC MRI and benchtop experiments at CO = 2 LPM for the LPA stenosis of varying severity. **A** CDP_LPA_, and **B** ${\overline{{\boldsymbol{E}}} }_{{\boldsymbol{l}}{\boldsymbol{o}}{\boldsymbol{s}}{\boldsymbol{s}},{\boldsymbol{L}}{\boldsymbol{P}}{\boldsymbol{A}}}$

The variation in CDP_LPA_ with varying degrees of LPA stenosis severity is plotted in Fig. 6A. The CDP_LPA_ increased with the LPA stenosis severity as observed from the 2D PC MRI results. This trend was similar to that observed in the benchtop results. At a 2 LPM CO, the CDP_LPA_, computed using 2D PC MRI, increased from 11.0 to 34.6 when the stenosis severity increased from 70% AS to 80% AS (Table 6). When the stenosis severity increased to 90% AS, the 2D PC MRI-based CDP_LPA_ increased to 154.2 (Table 6). These (2D PC MRI) values of CDP_LPA_ were consistently lower (absolute difference within 107) than the benchtop values (Fig. 6A and Table 6).

As observed from Fig. 6A and Table 7, the 3D PC MRI-based CDP_LPA_ was 11.5 for 70% AS, and compared well with the benchtop (absolute difference = 3) and 2D PC MRI (absolute difference = 0.5) values. Importantly, the CDP_LPA_ computed by all three methods (i.e. benchtop, 2D PC MRI, and 3D PC MRI) showed distinct and non-overlapping ranges for each stenosis severity (70% AS: 11.0 – 14.5; 80% AS: 34.6 – 60.7; and 90% AS: 154.2 – 261.6).

**Table 7:** Diagnostic parameters obtained with 3D PC MRI, 2D PC MRI and the benchtop flowmeter.

LPA stenosis severity	Modality	CDP_LPA_	${\overline{E} }_{loss, LPA}$ (mJ per ${Q}_{LPA}$)
70% AS	Benchtop	14.5	− 501.9
	2D PC MRI	11.0	− 434.6
	3D PC MRI	11.5	− 375.8

### Normalized Energy Loss (${\overline{{\boldsymbol{E}}} }_{{\boldsymbol{l}}{\boldsymbol{o}}{\boldsymbol{s}}{\boldsymbol{s}},{\boldsymbol{L}}{\boldsymbol{P}}{\boldsymbol{A}}}$)

The variation in ${\overline{{\boldsymbol{E}}} }_{{\boldsymbol{l}}{\boldsymbol{o}}{\boldsymbol{s}}{\boldsymbol{s}},{\boldsymbol{L}}{\boldsymbol{P}}{\boldsymbol{A}}}$ with varying degrees of LPA stenosis severity is presented in Fig. 6B and Table 6. As observed from the 2D PC MRI results, the ${\overline{{\boldsymbol{E}}} }_{{\boldsymbol{l}}{\boldsymbol{o}}{\boldsymbol{s}}{\boldsymbol{s}},{\boldsymbol{L}}{\boldsymbol{P}}{\boldsymbol{A}}}$ (absolute) generally increased with the increase in LPA stenosis severity, except for 90% AS (marked with an asterisk in Fig. 6B). At CO = 2 LPM, the ${\overline{{\boldsymbol{E}}} }_{{\boldsymbol{l}}{\boldsymbol{o}}{\boldsymbol{s}}{\boldsymbol{s}},{\boldsymbol{L}}{\boldsymbol{P}}{\boldsymbol{A}}}$ (absolute), computed using 2D PC MRI, increased from 434.6 mJ per ${{\boldsymbol{Q}}}_{{\boldsymbol{L}}{\boldsymbol{P}}{\boldsymbol{A}}}$ to 945.0 mJ per ${{\boldsymbol{Q}}}_{{\boldsymbol{L}}{\boldsymbol{P}}{\boldsymbol{A}}}$ (Table 6) when the stenosis severity increased from 70% AS to 80% AS. These values compared well (absolute difference within 303 mJ per ${{\boldsymbol{Q}}}_{{\boldsymbol{L}}{\boldsymbol{P}}{\boldsymbol{A}}}$) with the benchtop results (Table 6). An ${\overline{{\boldsymbol{E}}} }_{{\boldsymbol{l}}{\boldsymbol{o}}{\boldsymbol{s}}{\boldsymbol{s}},{\boldsymbol{L}}{\boldsymbol{P}}{\boldsymbol{A}}}$ (absolute) of 932.5 was obtained for the 90% AS using 2D PC MRI, which resulted in a higher absolute difference of 1002 mJ per ${{\boldsymbol{Q}}}_{{\boldsymbol{L}}{\boldsymbol{P}}{\boldsymbol{A}}}$ with the benchtop results (Table 6). The ${\overline{{\boldsymbol{E}}} }_{{\boldsymbol{l}}{\boldsymbol{o}}{\boldsymbol{s}}{\boldsymbol{s}},{\boldsymbol{L}}{\boldsymbol{P}}{\boldsymbol{A}}}$ (absolute) value obtained using 2D PC MRI for the 90% AS was lower than the value obtained for the 80% AS (Fig. 6B). The discussion section provides more insight into this finding.

The 3D PC MRI-based ${\overline{{\boldsymbol{E}}} }_{{\boldsymbol{l}}{\boldsymbol{o}}{\boldsymbol{s}}{\boldsymbol{s}},{\boldsymbol{L}}{\boldsymbol{P}}{\boldsymbol{A}}}$ (absolute) for 70% AS was 375.8 (Fig. 6B and Table 7), which was lower in comparison to the benchtop (absolute difference = 126.1) and 2D PC MRI (absolute difference = 58.8) values, as observed from Fig. 6B. More importantly, each stenosis severity was well-delineated by distinct and non-overlapping ${\overline{{\boldsymbol{E}}} }_{{\boldsymbol{l}}{\boldsymbol{o}}{\boldsymbol{s}}{\boldsymbol{s}},{\boldsymbol{L}}{\boldsymbol{P}}{\boldsymbol{A}}}$ values, except for the 2D PC MRI value for 90% AS.

## Discussion

The *in vitro* experiments involving the use of an MCL, subject-specific phantoms, and PC MRI provide a controlled environment for non-invasive diagnosis of peripheral pulmonary stenosis. Our recently published benchtop study [24] provided pathophysiologic hemodynamic data for designing the MRI experiments and assessing the importance of the MRI-based diagnostic technique.

With the advent of additive manufacturing and improved MRI technology, phantom-based PC MRI experiments have gained increasing importance among researchers for non-invasive assessment of vascular hemodynamics in a pre-clinical setting [18, 35–40]. In particular, extensive *in vitro* research for improving 3D PC MRI (or 4D MRI) technology has been conducted over the past few years using vascular phantoms. Most studies compute the velocity and flow field within the vessel from the phase and magnitude images for assessing indices such as peak (jet) velocity, retrograde flow, and stroke volume [41]. A few studies have extended the application of PC MRI to derive the pressure gradient across the vessel or region of interest using either the modified/simplified Bernoulli equation for 2D PC MRI data or the Navier-Stokes equation for 3D PC MRI data [18, 20, 37]. Pressure and flow rate data obtained from the phantom-based MRI scans have been mostly used as a baseline measure to assess MRI data from human subjects. However, comparison of pressure-flow rate data, obtained from MRI experiments, and direct measurements using pressure and flow sensors in phantoms have rarely been reported. Although phantom-based PC MRI experiments have shown promise in early feasibility studies, there are inherent challenges in obtaining a) a high signal-to-noise ratio, and b) a meaningful MR signal in the absence of realistic blood flow and vessel properties.

For the current study, system modifications such as a) the use of saline bags around the phantom, b) tuning of the velocity encoding value (VENC), and c) centralizing the location of the phantom within the magnet bore were necessary to acquire an MR signal with a moderate to high signal-to-noise ratio [42]. The comparison of flow rates computed using PC MRI (hereafter referred to as ‘PC MRI flow rates’) with those recorded directly by the flowmeter provided an indirect measure of the quality of the MRI data. Although the relative error in flow rate between the PC MRI and flowmeter measurements was relatively high (closer to 20%) for few cases, it is important to note that such a disparity is a consequence of inherent variabilities in both measurement techniques. For example, the flow rates recorded by the flowmeter were observed to fluctuate ± 200 ml/min of the value reported in Tables 4 and 5 for each experiment. Similarly, the PC MRI flow rates represent the statistical mean of three datasets (number of signal averages, NSA = 3) acquired by the scanner for each scan sequence during a specific experiment. Thus, the standard deviation in PC MRI measurements remains unknown because the resultant image dataset contains only the statistical mean velocity at each point in 2D or 3D space.

The quality of the MR signal may also be impacted by complex flow phenomena such as high-velocity jet, flow separation and recirculation, and shear layer instabilities occurring in the presence of a stenosis. Such flow phenomena have been known to cause imaging artifacts and signal loss, especially in the throat and distal regions of the stenosis, resulting in erroneous velocity and flow rate measurements [35, 38, 39]. O’Brien *et al*. [35] reported errors in flow rate measurements between PC MRI and the flowmeter ranging from less than 10% to greater than 50% in the presence of a stenosis, where the error increased with flow rate and echo time. Compared to this study, the errors (within 20%) in the present study were observed to be near the lower limit of the error range.

The error in PC MRI flow rates influences the pathophysiologic LPA flow split, which is an important parameter for evaluating the diagnostic indices. For example, the pathophysiologic ${Q}_{LPA}/{Q}_{MPA}$, derived from 2D PC MRI, was higher in case of the 80% AS and lower in the case of the 90% AS when compared to the benchtop results. These differences may have led to inconsistent trends in the total trans-stenotic pressure drop derived from PC MRI (= 3.49 mmHg (70% AS), 7.23 mmHg (80% AS), and 6.94 mmHg (90% AS)). Therefore, the lower ${\overline{E} }_{loss, LPA}$ (Fig. 6B) may be due to the lower pressure drop for 90% AS case. In addition to the effect of MRI signal quality, the estimation of trans-stenotic pressure drop from PC MRI data using simplified conservation laws may be another reason for the differences between the diagnostic parameter values computed from different techniques. For example, the pressure drop was derived using an unsteady laminar flow assumption, which is known to result in an underestimation of pressure drop of up to 18% in comparison to pulsatile flow calculations [32]. Therefore, the lower MRI-based CDP_LPA_ and ${\overline{E} }_{loss, LPA}$ values (Fig. 6), may be a consequence of the underestimated pressure drop.

Importantly, consistent trends were observed in the diagnostic parameters for the different severities of LPA stenosis, irrespective of the variations in the pathophysiologic LPA flow split. *Specifically, distinct and non-overlapping CDP*_*LPA*_* were observed for each case of LPA stenosis severity*. This trend in CDP is similar to those observed in *in vitro* [27–29] and *in vivo* [43, 44] studies on coronary artery stenoses. Such distinct CDP_LPA_ are achieved due to the high significance paid to the LPA flow (square of ${u}_{e,LPA}$) in the definition of CDP_LPA_. This fact allows CDP_LPA_ to have a better ability to delineate the stenosis severity compared to ${\overline{E} }_{loss, LPA}$, as reported in this study.

Further, this study demonstrates that PC MRI can be used a) as a non-invasive modality to quantify the pathophysiologic distribution of PA blood flow, and b) to derive hemodynamics-based indices for assessing PA stenosis in CHD patients. While this study has primarily focused on 2D PC MRI, the applicability of 3D PC MRI has also been explored. Although 3D PC MRI may be an improved technique, allowing the capture of transient velocity over the entire 3D flow domain, its accuracy and usefulness is limited by the use of a single VENC value over the entire scan volume. The VENC is typically selected to encompass the highest velocities within the flow domain. If the VENC is significantly higher than the lowest velocity in the flow domain, a low signal-to-noise ratio is observed, resulting in a less than optimum image quality. On the other hand, a VENC value that is set much lower than the highest velocity results in velocity aliasing.

As stated earlier, flow across the stenotic PA test section is highly complex and dynamic with appreciable spatial and temporal variations in the velocity field. Considering pathophysiologic flow at 2 LPM CO for the 70% AS case, the range in spatially-averaged velocity over a cross-sectional plane was observed to be 40 cm/s – 150 cm/s in the PA flow domain. The VENC value for this case was set to 130 cm/s during the 3D PC MRI data acquisition. This single value may not accurately capture the velocity in the regions with very low (i.e. in the MPA and recirculation regions distal to stenosis) or very high (i.e. in the stenosis throat and jet in the diverging region of stenosis) flow. Thus, the VENC needs to be optimized for each stenosis severity at a specific flow rate to obtain better quality 3D PC MRI data with minimal signal loss.

### Limitations of Pressure Drop Calculations

*Theoretical Equations*. The velocity data obtained from PC MRI was used as an input to mass and momentum conservation equations for deriving the pressure drop across the stenosis. This is an improvement over the modified/simplified Bernoulli’s equations for obtaining non-invasive pressure drop data and functional diagnostic parameters. The theoretical equations for pressure drop are derived using assumptions such as time-averaged unsteady laminar flow with shear layer instabilities, Newtonian fluid, parabolic velocity profile, and empirical constants for branch loss. Only theoretical equations were used to calculate ${\Delta p}_{MPA-LPA}$ as 1) invasive pressure measurements using pressure wires/transducers were not possible within the MR scanner, and 2) numerical transient calculations for pulsatile flow using CFD techniques were not the intended scope of this study. Therefore, previous studies in our and other labs [31–34] were used to discuss possible errors in ${\Delta p}_{MPA-LPA}$ calculations.

Time averaged unsteady laminar flow assumption is one of the limitations of the adopted analytical approach which neglects time dependent phenomena such as transient shear layer growth in the throat section and time varying recirculation zone distal to PA stenosis. Complexities associated with temporal variation with developing flow and non-Newtonian fluid can be evaluated as a future step. On a similar note, the velocity obtained from PC MRI represents an average value over specific cross-sectional planes placed proximal and distal to the stenosis. Therefore, the local variation in velocity within the flow domain may not have been fully captured.

The translesional pressure drop obtained through the analytical approach is, in general, lower than the ones from the numerical calculations using pulsatile flow. This variation in the pressure drop is due to simplified assumptions such as time-averaged unsteady flow and Newtonian fluid. However, this variation can be reduced by using a correction factor that can account for such limitations. In our previous studies on the translesional pressure drop for moderate and severe stenoses [45, 46], the ratio of the pressure drop for pulsatile and time-averaged unsteady flow condition is determined to be approximately 1.16. The analytical pressure drop can be multiplied by this factor to obtain the corrected pressure drop, and therefore, both CDP_LPA_ and ${\overline{E} }_{loss, LPA}$ can be re-calculated. The corrected pressure drops will further reduce the difference between the corresponding corrected analytical and the benchtop experimental [24] values or numerical calculations using pulsatile flow, once conducted in-future.

Previous studies [32, 46, 47] have shown that mean translesional pressure drop for severe (90%) PA stenosis can be obtained within 2–18% error of pulsatile calculations (also discussed above). It should be mentioned that due to the smaller magnitude of pressure drop for the moderate (70%) PA stenosis the percentage difference between numerical and analytical results could be relatively higher as compared to the intermediate (80%) and severe (90%) PA stenosis. However, it is expected that the higher percentage difference for moderate stenosis will not affect diagnostic assessments influencing clinical treatment as the magnitude of the pressure drop is smaller. Therefore, for intermediate to severe PA stenosis, time-averaged unsteady laminar assumption can provide valuable information on hemodynamic parameters such as pressure drop under clinical settings.

An alternate method has been proposed by our group [20] for non-invasively deriving pressure drop using 3D PC MRI. The method involves the use of streamlines, computed from the captured 3D velocity field, to derive the pressure drop across the stenosis at each time point in the cardiac cycle. The resultant pressure drop across the stenosis is an integration of the incremental pressure drops computed along streamlines; thus, accounting for the velocities at each location in the 3D flow domain. Therefore, the alternate 3D PC MRI methodology for calculating trans-stenotic pressure drop may provide an improved assessment of CDP_LPA_ and ${\overline{E} }_{loss, LPA}$. Furthermore, to enable widespread clinical adoption, a prospective clinical trial with a larger patient population across a range of CHD patients is required.

## Conclusions

An MCL in conjunction with a subject-specific vascular phantom was useful in generating pathophysiologic hemodynamics of a unilateral LPA stenosis in an *in vitro* setting. 2D and 3D PC MRI was used to acquire non-invasive pressure-flow data within the vascular phantom, and subsequently compute CDP_LPA_ and ${\overline{{\boldsymbol{E}}} }_{{\boldsymbol{l}}{\boldsymbol{o}}{\boldsymbol{s}}{\boldsymbol{s}},{\boldsymbol{L}}{\boldsymbol{P}}{\boldsymbol{A}}}$ for the three stenosis severities. To evaluate the efficacy of the MRI-based diagnostic method, the MRI results were compared with direct pressure-flow measurements obtained from a previous benchtop experimental study.

Overall, CDP_LPA_ and ${\overline{{\boldsymbol{E}}} }_{{\boldsymbol{l}}{\boldsymbol{o}}{\boldsymbol{s}}{\boldsymbol{s}},{\boldsymbol{L}}{\boldsymbol{P}}{\boldsymbol{A}}}$ (absolute value) showed an increasing trend with an increase in stenosis severity. More importantly, CDP_LPA_ showed distinct and non-overlapping values for each of the three stenosis severities when computed using all modalities (i.e., 2D and 3D PC MRI and benchtop experiments). Thus, CDP_LPA_ demonstrated superior diagnostic capabilities over ${\overline{{\boldsymbol{E}}} }_{{\boldsymbol{l}}{\boldsymbol{o}}{\boldsymbol{s}}{\boldsymbol{s}},{\boldsymbol{L}}{\boldsymbol{P}}{\boldsymbol{A}}}$ in delineating a unilateral LPA stenosis severity. Further, the pre-clinical evaluations using *in vitro* experiments show that PC MRI may be a comprehensive and safe clinical modality for the non-invasive diagnosis of branch PA stenosis. Future research required to standardize the use of the non-invasive diagnostic methodology for congenital PA stenosis include: a) enhanced and more accurate computations of the diagnostic parameters using 3D PC MRI and b) the use of a clinical study involving human subject data to evaluate CDP_LPA_ and ${\overline{{\boldsymbol{E}}} }_{{\boldsymbol{l}}{\boldsymbol{o}}{\boldsymbol{s}}{\boldsymbol{s}},{\boldsymbol{L}}{\boldsymbol{P}}{\boldsymbol{A}}}$ with PC MRI.

## Figures and Tables

**Fig. 7:**
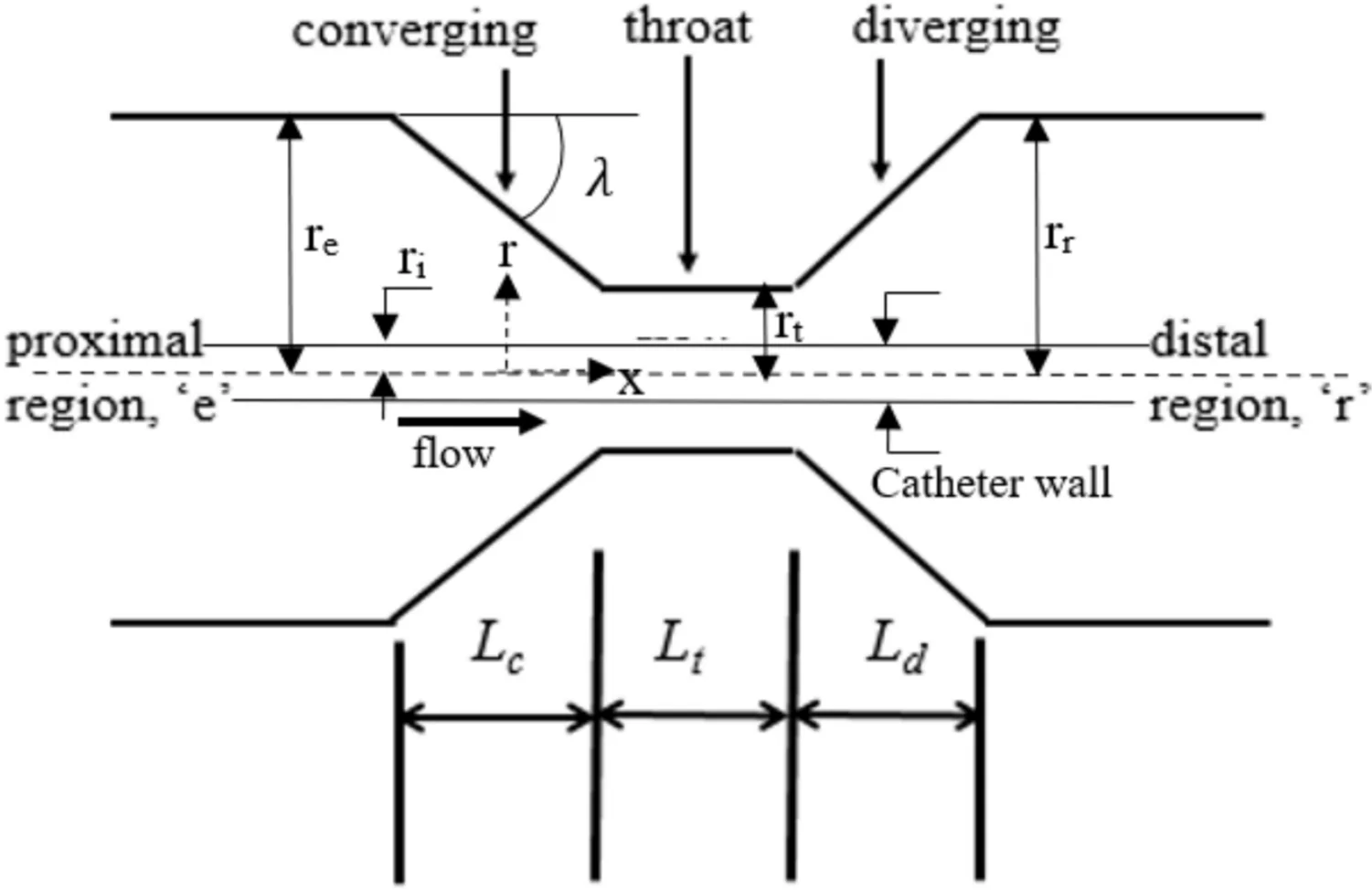
Geometry of stenosis; showing converging, throat, and diverging regions with catheter (or guidewire) placed concentrically

## Data Availability

The datasets used and/or analyzed during the current study are available from the corresponding authors on reasonable request.

## Derivation of the pressure drop across the converging section of the stenosis

See Fig. 7.

Equation 1a for the current study is derived from a generalized pressure–flow relationship formulated for a stenotic geometry that includes a catheter (or guidewire) (Fig. A1; [31, 32]). The generalized pressure-flow relationship is modified by removing the catheter/guidewire (${r}_{i}\to 0$) leading to equation 1a for this study. The derivation for the current study is described below.

The conservation of mass and momentum equations in their integral form are applied to find the relation between pressure drop, flow, and lesion geometry having a catheter (or guidewire) placed concentrically.

Mass balance equation:

A1 $$\frac{\partial }{\partial t}\int \rho dV+\int \rho \overrightarrow{V}dA=0$$

Momentum balance equation:

A2 $$\frac{\partial }{\partial t}\int \rho \overrightarrow{V}dV+\int \rho \overrightarrow{V}(\overrightarrow{V}.dn)=\sum F$$

After applying these equations to the geometry shown in Fig. A1, mass and momentum balance equation can be written as follows:

A3 $$\rho \frac{\partial A}{\partial t}dx+\rho d\int udA=0$$

A4 $$\sum F=-Adp-\left(2\pi {r}_{i}{\tau}_{wi}+{P}_{o}{\tau}_{wo}\right)dx=\rho d\int {u}^{2}dA+\rho \frac{\partial Q}{\partial t}\partial x$$

The mean momentum balance equation can be obtained by integrating equation (A4) over a period T of cardiac cycle

A5 $$-d\widetilde{p}=\frac{1}{A}\left(2\pi {r}_{i}{\widetilde{\tau }}_{wi}+2\pi {r}_{o}{\widetilde{\tau }}_{wo}\right)dx+\rho d\left(\beta \frac{{\overline{u} }^{2}}{2}\right)$$

where $\overline{u }$ is the average axial velocity during the cycle, ${r}_{i}$ is the catheter (or guidewire) radius, ${r}_{o}$ is the mean radius, and $\beta =\frac{1}{{\overline{u} }^{2}A}\int {u}^{2}dA$ is the energy correction factor based on the linear flow theory. The symbol (~) represents the time average of the corresponding parameters which will be dropped from the rest of equations in the paper. The mean pressure drop (described above) along the stenosis is obtained under steady, laminar flow, and Newtonian fluid assumptions.

Integrating equation A5 along the converging length results in the mean pressure drop correlation across this region [31, 32]:

A6 $${\Delta p}_{c}=\frac{128\mu {L}_{c}Q{I}_{s}}{\pi {d}_{e}^{4}}+\beta \frac{1}{2}\rho {u}_{e}^{2}\left[{\left(\frac{{A}_{e}}{{A}_{t}}\right)}^{2}-1\right]$$

where ${I}_{s}={\int}_{0}^{1}\frac{H}{{\left(\frac{{d}_{o}}{{d}_{e}}\right)}^{4}\left[1-{\left(\frac{{d}_{i}}{{d}_{o}}\right)}^{2}\right]}\left(1+\frac{{f}_{i}}{{f}_{o}}\right)d\left(\frac{x}{{L}_{c}}\right)$ is shear force integral with H defined as

$H=\left[1-\frac{\frac{1}{2}\left[1-{\left(\frac{{r}_{i}}{{r}_{o}}\right)}^{2}\right]}{\mathrm{ln}\left(\frac{{r}_{o}}{{r}_{i}}\right)}\right]F$, while $\frac{{f}_{i}}{{f}_{o}}$ is defined as

$$ \frac{{f_{i} }}{{f_{o} }} = \frac{{{\raise0.7ex\hbox{${{2}\pi {\mathrm{r}}_{i} }$} \!\mathord{\left/ {\vphantom {{{2}\pi {\mathrm{r}}_{i} } {\tau_{wi} }}}\right.\kern-0pt} \!\lower0.7ex\hbox{${\tau_{wi} }$}}}}{{{\raise0.7ex\hbox{${{2}\pi {\mathrm{r}}_{o} }$} \!\mathord{\left/ {\vphantom {{{2}\pi {\mathrm{r}}_{o} } {\tau_{wo} }}}\right.\kern-0pt} \!\lower0.7ex\hbox{${\tau_{wo} }$}}}} = \frac{{ - \left( {\frac{{r_{i} }}{{r_{o} }}} \right)^{{2}} + \frac{{\frac{{1}}{{2}}\left[ {{1} - \left( {\frac{{r_{i} }}{{r_{o} }}} \right)^{{2}} } \right]}}{{\ln \left( {\frac{{r_{o} }}{{r_{i} }}} \right)}}}}{{{1} - \frac{{\frac{{1}}{{2}}\left[ {{1} - \left( {\frac{{r_{i} }}{{r_{o} }}} \right)^{{2}} } \right]}}{{\ln \left( {\frac{{r_{o} }}{{r_{i} }}} \right)}}}} $$

In these equations ${r}_{o}$ varies with axial length $\left(x\right)$ as ${r}_{o}={r}_{e}-x\mathrm{tan}\left(\lambda \right)$, where $\lambda ={\mathrm{tan}}^{-1}\left(\left({r}_{e}-{r}_{t}\right)/{L}_{c}\right)$ is the slope of converging section and x is calculated from the stenosis leading edge as shown in Fig. A1. In the absence of catheter/guidewire (as in present study), ${r}_{i}\to 0$, F→1 and H→1, thus reducing the shear force integral as: ${I}_{s}={\int}_{0}^{1}\frac{{r}_{e}^{4}}{{\left[{r}_{e}-\frac{x}{{L}_{c}}\left({r}_{e}-{r}_{t}\right)\right]}^{4}}d\left(\frac{x}{{L}_{c}}\right)$. It can be written in terms of diameter as ${I}_{s}={\int}_{0}^{1}\frac{{d}_{e}^{4}}{{\left[{d}_{e}-\frac{x}{{L}_{c}}\left({d}_{e}-{d}_{t}\right)\right]}^{4}}d\left(\frac{x}{{L}_{c}}\right)$, where d_e_ = 2r_e_, and d_t_ = 2r_t_.

## Abbreviations

2D: Two-dimensional
3D: Three-dimensional
4D: Four-dimensional
CHD: Congenital heart disease
PA: Pulmonary artery
MPA: Main PA
LPA: Left PA
RPA: Right PA
RV: Right ventricle
CO: Cardiac output
CT: Computed tomography
MRI: Magnetic resonance imaging
CMR: Cardiac MR
PC: Phase contrast
CAD: Computer-aided design
MCL: Mock circulatory loop
BAF: Blood analog fluid
AS: Area stenosis
CDP: Pressure drop coefficient
${\overline{E} }_{loss}$: Normalized energy loss
VENC: Velocity encoding (or aliasing velocity)
TE: Echo time
TR: Repetition time
d_e_: Proximal diameter
d_r_: Distal diameter
d_t_: Throat diameter
d_i_: Catheter diameter
d_o_: Mean diameter

## Declarations

### Citation Diversity Statement

Recent work in several fields of science has identified a bias in citation practices such that papers from women and other minority scholars are under cited relative to the number of papers in the field. We recognize this bias and have worked diligently to ensure that we are referencing appropriate papers with fair gender and racial author inclusion.
